# SCC*mec* Typing in Methicillin-Resistant *Staphylococcus aureus* Strains of Animal Origin

**DOI:** 10.3201/eid1501.071647

**Published:** 2009-01

**Authors:** Marc D. Jansen, Adrienne T.A. Box, Ad C. Fluit

**Affiliations:** University Medical Center Utrecht, Utrecht, the Netherlands

**Keywords:** Staphylococcus aureus, SCCmec, animal, letter

## To the Editor

Van Loo et al. described the presence of staphylococcal cassette chromosome *mec* (SCC*mec*) type III in some methicillin-resistant *Staphylococcus aureus* sequence type (ST) 398 isolates related to pig farming (*1*). SCC*mec* types are based on the allotype of *ccr* genes and the *mec* gene complex. Class A *mec* has intact *mecI/R* regulator genes. Type III SCC*mec* has type 3 *ccr* genes and class A *mec* complex, whereas type V SCC*mec* contains *ccrC* and class C *mec* (*2**,**3*). The authors typed SCC*mec* of the isolates by the method of Zhang et al. (*4*), in which type III is defined by amplification of a 280-bp fragment located in the junkyard region. This fragment is found in SCC*mer* that is associated with SCC*mec* type III.

We have typed SCC*mec* of the same 4 isolates that were reported to be SCC*mec* type III positive by using the primer sets defined by Ito et al. (*2*,*3*) and Lim et al. (*5*) for *ccr* types 1–3 and *ccrC* and 4 additional primers developed at our institute (Table) in single PCRs. All ST398 isolates were PCR negative when primers specific for SCC*mec* type III were used, but PCR positive with the *ccrC*-specific primers. DNA sequencing confirmed the product as *ccrC*. Further, the isolates did not have a class A *mec* complex, a requisite for SCC*mec* type III, because a *mecI*-specific PCR was negative for these isolates. In addition, Southern hybridizations with digoxigenin-dUTP–labeled PCR fragments obtained with our primer pair specific for *ccr3* and primers for *ccrC* (*3*) showed no hybridization with the *ccrA/B3* probe (except for the positive control). All of the ST398 isolates hybridized with the *ccrC*-specific probe.

**Table:** Primers used to type SCC*mec* of MRSA ST398 isolates*

Genes	Primer name	Primer sequence (5′ → 3′)
*ccrA/B*1	ccr1B-for	CTT TCA CGA TAG ACA CAG
	ccr1B-rev	TAA AAG AAG TTC ATA GCC GTT AAA TTG G
*ccrA/B*2	ccr2B-for	GCA TTC ATC ATC AAT CAA AAT G
	ccr2B-rev	CTA TAA CCT TCT GTG CTT TGC A
*ccrA/B*3	ccr3B-for	TCC GTA ATA AGA AGC AAC TTC AC
	ccr3B-rev	ACT ATA GCC TTC AGT ACT TTG GA
*ccrA/B*4	ccr4B-for	TGA AGA AGC ACA AGA GCG GC
	ccr4B-rev	CTG CAC CAC ATT TTG GGC AC

*SCC*mec*, staphylococcal cassette chromosome *mec*; MRSA, methicillin-resistant *Staphylococcus aureus*; ST, sequence type.

We conclude that on the basis of generally accepted definitions SCC*mec* type V is present in these ST398 pig-farming–related isolates, not SCC*mec* type III. Therefore, researchers should be aware that some typing methods may lead to inadequate results.
