# Supplementary material for: SCCmec Typing in Methicillin-Resistant Staphylococcus aureus Strains of Animal Origin
Source: Emerg Infect Dis. 2009 Jan;15(1):136–7. doi: 10.3201/eid1501.071647 (PMC2660682; doi:10.3201/eid1501.071647)
Supplement: Related texts

# Reply: In Response

**Authors:** Xander Huijsdens, Inge van Loo, Jan Kluytmans

**Affiliations:** Institute for Public Health and the Environment, Bilthoven, the Netherlands (X. Huijsdens); University Hospital Maastricht, Maastricht, the Netherlands (I. van Loo); Amphia Hospital, Breda, the Netherlands (J. Kluytmans); Medical Microbiology and Infection Control, VU Medisch Centrum, Amsterdam, the Netherlands (J. Kluytmans)

We thank Jansen et al. for their comments about the SCC*mec* types of sequence type (ST) 398 methicillin-resistant *Staphylococcus aureus* (MRSA) isolates (*1*). For SCC*mec* typing of MRSA, several different PCR methods have been published. We originally chose the SCC*mec* PCR developed by Zhang et al. (*2*) because at that time it was the method of choice in many published papers. Fluit et al. questioned whether the SCC*mec* type III isolates were correctly typed (*1*). To prove that the results of typing these 4 isolates were incorrect, these researchers performed several different SCC*mec* PCRs, including a PCR with primers they developed themselves. In addition, Southern hybridization was done. The results showed that SCC*mec* III ST398 MRSA isolates should be typed as SCC*mec* type V. In this conclusion we agree with the authors. It seems clear that Zhang’s method incorrectly identified 4 of the animal-related ST398 isolates as SCC*mec* type III instead of SCC*mec* type V. Whether all ST398 MRSA are SCC*mec* type IV or V remains unclear. Recently, an article by Nemati et al. was published in which ST398 MRSA was also typed as SCC*mec* III (*3*). However, in that study the SCC*mec* typing method of Zhang was also used.

In conclusion, the choice of SCC*mec* typing method is directly related to obtaining accurate SCC*mec* results for ST398 isolates. To date, almost all animal-related ST398 MRSA isolates are SCC*mec* types IV and V.

## References

[1] Jansen MD, Box ATA, Fluit AC. SCC*mec* typing in methicillin-resistant *Staphylococcus aureus* strains of animal origin. Emerg Infect Dis. 2009;15:136. 10.3201/eid1501.07164719116081PMC2660682

[2] Zhang K, McClure JA, Elsayed S, Louie T, Conly JM. Novel multiplex PCR assay for characterization and concomitant subtyping of staphylococcal cassette chromosome *mec* types I to V in methicillin-resistant *Staphylococcus aureus.* J Clin Microbiol. 2005;43:5026–33. 10.1128/JCM.43.10.5026-5033.200516207957PMC1248471

[3] Nemati M, Hermans K, Lipinska U, Denis O, Deplano A, Struelens M, Antimicrobial resistance of old and recent *Staphylococcus aureus* isolates from poultry: first detection of livestock-associated methicillin-resistant strain ST398. Antimicrob Agents Chemother. 2008;52:3817–9. 10.1128/AAC.00613-0818663024PMC2565892
